# Context sensitive mindfulness: lessons from graduates of a professional training in South Africa

**DOI:** 10.1186/s12906-025-04775-4

**Published:** 2025-02-26

**Authors:** Simon Whitesman, Robert Mash

**Affiliations:** Division of Family Medicine & Primary Care, Stellenbosch University, Francie Van Zijl Drive, Parow, Cape Town, 7505 South Africa

**Keywords:** Mindfulness, Mindfulness-based interventions, Trauma-sensitive mindfulness, Context sensitive adaptations, Continuous traumatic stress, Holding environment, Compassion, South africa

## Abstract

**Objective:**

The study aimed to evaluate the implementation of mindfulness based interventions, in different community settings in South Africa, by graduates of a two-year mindfulness training course, and to explore the challenges involved in adapting to the local context.

**Methods:**

This was a descriptive exploratory qualitative study using semi-structured interviews. Ten graduates of a two-year training in mindfulness-based interventions (MBIs) were identified using purposeful criterion-based sampling based on their implementation of adapted MBIs in communities that represented the systemic social, economic and health challenges affecting a majority of South Africans.

**Results:**

Previous and ongoing trauma is pervasive in South Africa which significantly affects the quality of lived experience for many individuals and families. Teachers who offered mindfulness-based approaches within these communities needed to hold a high level of sensitivity to pre-existing and ongoing trauma and signs of traumatic abreaction to effectively and skilfully deliver these interventions. Context sensitive adaptations needed to be implemented to programme structure, such as length of sessions, prioritisation of curricular elements and duration of mindfulness practices, along with in-the-moment flexible responsiveness such as ending a formal practice ealier than planned, or responding to the emotional needs of an individual. This was supported by the creation of a robust and compassionate holding environment, a safe and secure space in which attuned relationality supported co- and self-regulation and the internalisation of mindfulness skills.

**Conclusion:**

Mindfulness can be a valuable practice in diverse settings in South Africa, including communities affected by previous and current trauma, and the training curriculum in this context requires high levels of sensitivity to these conditions and must prioritise a safe and compassionate environment in which to learn.

## Introduction

Mindfulness based interventions (MBIs) are being increasingly adapted to different contexts and modes of delivery. There are many adaptations, with variability in their evidence base, from pilot studies with little scale-up, to programmes with an extensive research base and wide implementation, to those with a wide uptake, but little to no evidence [1]. Mindfulness-based stress reduction, as the original MBI, provides the foundation from which increasingly granular adaptations may evolve. These adapted forms serve a wider range of people: clinical populations, such as those with recurrent depression [2] or substance use disorder [3, 4]; different demographics, such as young adults [5] or expectant parents [6]; different modes of delivery, mainly through on line offerings as well as apps [7–9]; and qualitative shifts in emphasis such as compassion-centered programmes [10].

The recent literature provides insights on the utilisation and benefits of MBIs in racial minority groups in high-income countries, predominantly the United States [11, 12], while contextually sensitive adaptations are considered in South America [13] and Israel [14]. There is limited work within South Africa for specific groups such as people with HIV and refugees [15, 16]. Constraints on the transferability of this work are related to systemic and cultural issues, intergenerational trauma and lower socio-economic status. These issues are increasingly incorporated into adapted MBIs in various minority communities [17–21]. It remains an unaddressed question whether this new understanding and approaches to teaching would be the same in countries where such minority groups are the majority. There is much to be learned from the evidence emerging from high-income countries; however, the evidence-base for MBIs in low- or middle-income countries (LMICs) is as yet unclear.

While MBIs have been offered in South Africa since 1999 [22], there is very limited access in the broader African context to these interventions. We have previously reported the experience of, and effects on, professionals going through a training in MBIs in South Africa, a middle income country [23–25]. Teachers-in-training internalised the essential qualities of mindfulness as an awareness practice, as evidenced by an increase in mindfulness and self-compassion scores, and self-reported enhanced self-awareness. This required the effort of personal practice alongside theoretical understanding, the support of a learning community and a willingness to be present with a wider range of experiences. This was particularly significant when navigating challenging emotional and somatic experiences. These factors combined to support the embodiment of the essential qualities of mindfulness and the personal shifts that they experienced provided a robust foundation on which to begin teaching a contemporary mindfulness approach to others in a context-sensitive way.

The current study evaluated the issues involved in adapting MBIs in different settings in South Africa by graduates of this two-year university-level training [25]. This should provide insights into the curricular elements amenable to adaptation [26] and enhance its relevance, applicability and value in the local context. There was particular focus on those graduates who were teaching in impoverished or disadvantaged communities, which involved cross-cultural, linguistic, gender and socio-economic factors, rather than a narrow band of privilege. These diverse contexts reflected the socio-economic and lived realities of a majority of people in this country, in order to better prepare future students to teach mindfulness in these contexts.

## Method

### Study design

This was a descriptive exploratory qualitative study using semi-structured interviews with the teachers of MBIs.

### Setting

South Africa has been a constitutional democracy since the fall of Apartheid in 1994. It is a multi-cultural and -linguistic population of approximately 60 million, and is considered a high-middle income country. South Africa has some of the highest global ratings for economic inequity [27], rates of HIV [28], tuberculosis [29], foetal alcohol syndrome [30], inter-personal violence, especially femicide [31], unemployment [32], and levels of illiteracy or innumeracy [33]. South Africans also suffer high rates of mental health issues based on adverse childhood experiences and low socio-economic status [34, 35].

Approximately 160 people completed the training programme by the end of 2021, with 71% teaching an MBI after graduation [25]. The training consisted of four modules, structured as a series of 8–10 week short courses. The curriculum used mindfulness-based stress reduction (MBSR) as a foundation, with a strong emphasis on group learning and sensitivity to the context. The training programme has been fully described in a previous publication [25]. All students were required to teach one 8-week MBI, while being supervised weekly, by the end of the fourth module. Supervision also supported graduates in the planning and implementation of adaptations to the communities where they were already working. Mindfulness-based interventions were offered in diverse settings, with a predominance in disadvantaged communities, mainly in urban areas. Table 1 describes the graduates, contexts and interventions in more detail.

**Table 1:** Graduates & their teaching contexts

Interviewee	Gender	Occupation	Year of graduation	MBI	Context	Illustrative quotation on context
G1	F	Counsellor	2017	Mindfulness-informed peer support programme	Refugee peer support counsellors Urban community-based non-profit organisation (NPO)	“We would find those refugee peer counsellors in the communities in which we were working. They have had journeys, terrible stories and then we would train them if they were agreeable, to actually run these groups. There were a lot of refugees from Rwanda and Burundi and those kind of countries with strong histories of war and then we also Zimbabweans and Malawians too, who were more economic. There was a lot of trauma coming into the work”
G2	M	Religious minister	2018	MBSR and mindfulness-informed retreats	Catholic community religious leaders and staff Urban faith-based community	“I am Catholic priest at the moment in the Diocese of Port Elizabeth and I’ve been a priest now for 18 years. I am teaching mindfulness in a context of a three month sabbatical for religious sisters and brothers, Catholic priests from Southern Africa. All they (attendees) said was the most important time or the most important thing that we did there was that mindfulness week”
G3	M	Psychiatrist	2016	Mindfulness-based stress reduction (MBSR) and Dialectical behavioural therapy (DBT)	Health professionals (MBSR) & In-patients (DBT) Secondary community psychiatric hospital	“The bulk of my work was with complex and severe mental illness and in particular complex trauma, complex post-traumatic stress (PTS) and borderline personality disorder”
G4	M	Coach/Mentor	2020	Adapted MBSR	Students at a coding school Urban, community-based training institution	“The majority of the students that I work with come from disadvantaged backgrounds, from the townships around Cape Town. I would say 70 or 80% black or even 90 depending on that years intake. I’ve worked mostly in the past couple of years with women who want to train to become software developers”
G5	F	Counsellor	2015	Mindfulness-informed parenting programme	Muslim women Urban, community-based parenting programme	“The parenting programmes were very much the parent centre’s programmes and then I came in with the mindfulness which filtered all the way through every session and included in home practice. We would start with a mindfulness practice but also with an Islamic prayer and the mindfulness practices really were interwoven through all the trainings. Very few of the women who attended had ever had the experience of being held in safety and being heard. It’s really quite remarkable”
G6	F	Community non-profit director	2018	Mindfulness-informed educational programme	Ealy childhood development (EDC) support workers Semi-rural, community-based EDC centre	“The concern of this organization was the fact that they are cared for by women who have no education so they might fall into the cracks because they are not supported by government. The women are not subsidized for the work that they do and they look after these children because they are their neighbour’s children and perhaps for a little income they will get from neighbours. Our concern was that whilst they are looking after these children, these kids are not stimulated in any way because the ladies do not have skills of stimulating children in their care”
G7	M	Religious minister	2020	MBSR	Church community members Urban, Gereformeerde Kerk (NGK)	“I am a minister in the Dutch Reformed Church in Moeder Kerk and part of a large group – we have six fulltime ministers and my focus is on families, parenting, marriages”
G8	M	Religious minister	2018	Introduction to mindfulness ; MBSR	Church community members On-line for Nederduitse Church community	“The one is church specific where I did it for the congregation and with the congregational context in a Christian languaging. Mindfulness can be a vehicle of opening their eyes to their own divinity and using a secular vehicle to enhance their lives”
G9	F	Coach	2020	MBSR	Female adolescents Urban, community-based support programme for (female) adolescents	“The group are from an informal settlement: they have no running water, they have no electricity, they use portable toilets, and also, every second house, there’s a shebeen there’s a tavern where you find men loitering all the time. So the context of the girls here is what I could call dire. “
G10	M	Religious minster	2018	Adapted MBI (with rolling intake)	Gangsters in recovery Urban, community, faith-based rehabilitation centre for recovering gangsters	“I wanted to work amongst the most violent people I can come across in the Cape Town area…and I found myself amongst the gangsters of Hanover park (in the Cape Flats, outside Cape Town). I never forgot my intention. I know what I am looking for. I am looking for the presence of God in the heart of evil…”

### Participants

Interviewees were graduates of the course, who completed their studies between three and eight years ago. Graduates were purposively selected, based on the first author’s (SW) knowledge of who had been teaching an adapted MBI in communities that reflected some of the social dynamics of the majority of South Africans, as described above. An additional consideration was to select people from different regions across South Africa. Graduates were approached until there was saturation of data in the last two interviews and no new themes emerging.

### Data collection

In-depth semi-structured interviews were conducted with an interview guide that included the following topics:

- The experience of the teachers with teaching in their context and how they adapted the MBI.
- The perspective of the teachers on how the two-year course prepared them for teaching MBIs within these contexts.
- The challenges of teaching in these contexts and how the they related to these through the lens of mindfulness.
- How they created a safe container for the group and the challenges in creating this.
- How participants engaged with and internalised mindfulness practice and how the teaching was adapted to enable this.

The interviewer was also open to explore other topics of relevance to the study aim. Interviews were conducted in 2022-23, on-line via Zoom, in English, by the first author (SW) and were recorded. Field notes were taken during the interviews. English was the language of the original course. Interviews lasted approximately 75–90 min.

### Data analyses

A professional transcriber created verbatim transcripts that were checked by the first author. The transcripts were then thematically analysed using the framework method, with the help of Atlas-ti software. The framework method was originally developed for thematic analysis of applied policy research [36] and has been extensively used within family medicine and primary care research in South Africa [37]. Analysis was performed by the first author (SW) and reviewed by the second author (RM), particularly the development of the coding index and interpretation of the data [38]:

1. Familiarisation: the researcher read the transcripts, revisited the audio recordings if necessary, reviewed any field notes and became familiar with the data. During this process the researcher noted issues emerging from the data that should be coded.
2. Coding index: Issues identified in step 1 were developed into codes. Codes were defined and organised into categories within a coding index. Codes were designed to be mutually exclusive, while covering all the issues that emerged in step (1) Both researchers reviewed and agreed on the final index. Codes were then created in Atlas-ti.
3. Coding: All transcripts were uploaded to Atlas-ti and coded using the index. Additional codes were created if necessary to capture emergent issues that were not identified in step 1.
4. Charting: Codes were then organised into code families within Atlas-ti that corresponded to the categories from step (2) Reports were created based on these families that included all the data related to these codes.
5. Interpretation: The reports were interpreted to identify key themes and subthemes, and to identify any relationships between themes. A random sample of four respondents validated the core themes.

### Trustworthiness

The first author of this paper (SW) was the convenor of the two-year training programme and knew all the graduates. He was also the curator of most of the curricular content and was committed to adapting mindfulness to the South African context. He is a medical doctor and psychotherapist who, along with a colleague, was the first professional to offer MBSR in South Africa [39]. He had previously conducted qualitative interviewing and analysis. Interviewees were encouraged to speak openly and honestly about their experience to offset an overly positive bias. Questions were deliberately open-ended and the tone of the interviews was non-judgmental and curious. The Consolidated Criteria for Reporting Qualitative research (COREQ), a 32-item checklist for interviews and focus groups, guided the reporting of the work [40].

## Results

Interviews were held with 10 graduates as shown in Table 1. Most interviewees were white-bodied, which aligns with the racial representation of students on the two-year training, but not the country. There were no refusals to participate or drop-outs. Participants included four women and six men, graduated between 2015 and 2020, and included a variety of professional backgrounds from a psychiatrist to coaches or counsellors and religious ministers. The MBIs included five MBSR-type programmes and five mindfulness-informed programmes. The focus of the MBIs was diverse and ranged from parents, adolescents, peer support counsellors, health professionals to refugees and gangsters. The contexts included urban, semi-urban and rural settings across three provinces, with most of the MBIs offered within existing organisations or institutions.

The findings are presented as six themes:

1. Trauma: previous and concurrent trauma and a trauma sensitive approach.
2. The holding environment: co-creation of a safe, robust, and compassionate learning and healing space.
3. Relationality: the centrality of relationality in new ways of being, seeing and relating.
4. Being embodied: the foundation of body awareness in teaching and practising mindfulness.
5. Regulation: the relationship and function of co- and self-regulation.
6. Adaptations to form, practice and process.

### Trauma: previous and concurrent trauma and a trauma sensitive approach

Respondents commented on the historical and concurrent social dynamics that characterised the contexts in which they delivered MBIs, in which people of colour were predominantly affected. They saw trauma as an ubiquitous phenomenon. In particular, they characterised many of these contexts as people with little education, unemployment, low income, and limited access to resources (e.g. electricity). In addition, refugees carried the traumatic impact of fleeing their homelands, along with the reasons precipitating such decisions:

*“High levels of unemployment that we’re seeing in our communities*,* high levels of school dropouts. So with that comes a high level of violence*,* of gender-based violence*,* of rape*,* of so many social inequalities and such a lot of trauma that you see in those areas. Some were living in perpetual domestic violence that they were witnessing each day.” (G9)*.

A common thread running through the narratives was the experience of trauma, particularly from interpersonal violence. Graduates were struck by the degree to which this had become normalised, a part and parcel of everyday life in impoverished communities, with limited awareness of how this was a recurring pattern across generations. Respondents reported that children witnessed and experienced physical abuse at the hands of their close relations, for example, and often repeated these behaviours when they grew up:

*“You will find that it’s a similar pattern*,* that you are dealing with people who have been so hurt that they didn’t have any other way of dealing with their hurt except to replicate it*. *If you grew up being beaten up*,* you think it’s the norm. Is there a way that we can unlearn that? So that was my first*,* you know*,* introduction*,* looking at how can we introduce mindfulness in this type of situation. How can we calm down the parents so that they begin to be aware of what is around them*,* what is happening.” (G6)*.

There was a high level of sensitivity to trauma amongst all the interviewees, even before offering a MBI. Many teachers described the value of introducing people to a taste of mindfulness prior to offering the intervention, through brief introductions to practice and some basic theory as most participants had no previous exposure. Graduates appreciated the potential for mindfulness to bring traumatic material into awareness, and given the pervasiveness of trauma in these communities, this approach enhanced receptivity to further exploration of the practice. For example, caregivers of adolescents were invited to attend an introductory session so the teacher could explain to them that when the girls were practising quietly at home, they were “not being lazy and not helping around the house”. It also informed the way they prepared the structure, presented the content and engaged with participants’ trauma experience, which was always informed by an understanding of the dynamics of individual and collective trauma. For example, when working with gangsters, the structure needed to accommodate a rolling intake as the programme was part of a rehabilitative facility in which men moved in and out of the unit fairly frequently. This required the teacher to incorporate the reality that some were completely new and others familiar with mindfulness, in almost every session. In working with early childhood development counsellors, content was often simplified to focus on a few aspects of mindfulness, such as short awareness of breath practices, rather than on offering too many different forms of practice. This often related to an understanding that trauma lives in the body, and that attitudes, such as compassion, needed to be introduced with a high degree of sensitivity:

*“ I learnt about self-compassion and going a step further*,* the loving kindness work …I needed to be much more careful than I realised*,* because what I’ve noticed again and again in this work*,* is going too hard down that road can provoke extreme anxiety where there’s a sense that I*,* in my childhood*,* did not get that or did not get enough of that. My experience in one of the groups was when we did a loving kindness meditation there was extreme dissociation*,* almost immediate and extreme anxiety and tearfulness. So*,* I’ve realised one needs to mitigate against that before introducing it too directly.” (G3)*.

### Holding environment: co-creation of a safe, robust, and compassionate learning and healing space

The environment in which mindfulness was taught needed to be containing, such that participants felt explicitly and implicitly held by the teacher in a safe and respectful way. All the factors, which contributed to this holding space, were embodied by the teachers in the initial stages of group formation and generalised to group members as the process unfolded. In certain contexts, ritual supported the creation of a safe learning environment. For example, the work with refugees was supported and contained through the lighting of two candles at the beginning of each session, to represent both the participants themselves and all those who they had lost and left behind. This meant that no one was left out of the process through the living symbol of a candle flame. Within religious communities, the use of a familiar biblical phrase supported connectedness amongst group members. For example, the Hebrew word “Hineni” from the book of Exodus - meaning “I am here” - was spoken by each participant at the beginning of every session as a way of landing in the space.

Attitudinal qualities, inherent to mindfulness, informed the interactions within the group. The quality of kindness was consistently described, in all contexts, as being a fundamental requirement. Various factors infused within this foundation were:

Acceptance, respect and honouring of participants’ experiences and backgrounds, engagement with interest and curiosity, consistency and reliability as traumatic environments were often characterised by chaos and unpredictability, such as availability of food. Teachers were intentionally reliable with how they showed up both structurally (consistent with their times and availability) and relationally (consistent with their care and attention):

*“I started every session (with the gangsters) with loving kindness: May I be safe. May I be this and that. So I started every session with that. And we ended with*,* ‘God loves me. I love myself. That is lekker (colloquial for nice/good).’ Ja*,* so that was very important.” (G10)*.

Teachers reminded participants that mindfulness is an inherent quality in all human beings, irrespective of context, history or narrative. It is an inner resource, that can be developed from the inside. Interviewees reported that, for many of their participants, recognising that they had access to a resource within themselves was empowering. Respondents worked skilfully to identify and reduce obstacles to practice. These included managing expectations (for example, they would not always feel an immediate benefit), being explicit about what mindfulness is and is not (it is not invariably relaxing) and what might occur in moments of silence (such as difficult feelings coming up):

*“I was trying to introduce something that you can call it very foreign to this particular audience and yet I thought it’s worth it. You are not asking them to buy anything*,* you are asking them to utilize what they are born with and to unlearn.” (G6)*.

Teachers perceived that, for many of their participants a willingness to express and share difficult feelings was diminished, and were often surprised that they had never had the experience of feeling safe enough to express themselves emotionally. One of the ways that many respondents supported the development of this capacity was through a willingness to be open and honest about their own feelings, including distressing ones, without being reactive. This implied remaining present and connected and implicitly invited other participants to explore expressing themselves in a similar way:

*“Bringing it back as a teacher*,* and making them see that I also am a human being who’s feeling*,* who’s sensitive.” (G9)*.

*“Having a teacher who models how you show up in the world and offers alternatives so that you know when you go off into your world you take that teacher with you and you try that out*,* you see what it feels like and it feels like it’s a much more supportive and wiser way of engaging.” (G5)*.

### Relationality: the centrality of relationality in new ways of being, seeing and relating

Creating a holding environment was a pre-condition for the generation of a relational field. The teacher, through their awareness of this central element, intentionally embodied and expressed qualities that invited mutuality, a sense of seeing and being seen in a dynamic, unfolding exchange. This included warmth, kindness, curiosity, acuity of listening and a willingness to share their own experiences in a real and, at times, vulnerable way:

*“Everything around the whole experience is relationship – it’s what you develop and what you offer and connect to and what they’re offering back. It’s the exchange that’s happening there.” (G1)*.

Another factor that helped develop relationships was the normalising of what emerged for participants, in their mindfulness practice and through the group enquiry, such as the universality of the wandering mind. By offering this perspective, teachers created an environment of non-judgment, which further supported the development of relationality.

The choice and skilful use of language was an important consideration, especially when the general language of instruction was not the mother tongue for many participants. Respondents described the value of being flexible with how and what language was used, when guiding a practice or doing an enquiry. For example, using words or phrases in isiXhosa or isiZulu in the middle of guiding a practice or enquiry in English. Teachers’ perception was that this offered participants an implicit sense of being understood and respected.

As relationships and authentic expression developed, respondents described how their participants appeared to feel increasingly seen, not only by the teacher, but also by others in the group. In this instance, what was seen and shared was the simple reality of each other’s human-ness, which often transcended role, social position or hierarchy:

*“It felt like they were just seeing each other as human beings…it’s like boundaries or walls just came down*,* like people just met each other for that session. There was just so much Ubuntu*,* which is being a human being*,* not Ubuntu as in that philosophical Ubuntu that we talk about which I think are two different things…it’s how we talk about it - humanness. The effects of that are quite striking: a lightening*,* an opening*,* a connecting*,* a sharing.”(G4)*.

### Regulation: the relationship and function of co- and self-regulation

Regulation informed and arose out of relationality, included both co- and self-regulation, and was infused with sensitivity to people’s trauma. This was a dynamic process, which was generally grounded in the teacher’s capacity to regulate their own experience. From this foundation, they supported individuals and the group to co-regulate, especially with respect to being lost in thoughts and difficult feeling states, as well as physiological hyper-arousal. Over the duration of a session or the whole course, teachers felt that participants began to internalise what they were learning with and through the teacher. This empowered them to develop the skill of self-regulation and to feel the effect for themselves:

*“I said*,* I close my eyes and is easier to do it with your eyes closed. But if you don’t want to*,* just look at the floor in front of you. And then*,* if anything happens… If you start getting frightened*,* upset or angry or something – just open your eyes and look at me. Okay. And just watch your breathing and we will be ready for that.” (G10)*.

The flexible utilisation of the breath was a practical and effective tool that supported regulation. Teachers often guided people to a momentary mindfulness of breathing as well as paced (regulated) breathing, such as a few deep breaths or lengthening the out-breath. This was perceived to be especially useful when participants were struggling with distressing physical and emotional states. Teachers were sometimes explicit in practising what they were guiding, as a way of modelling, encouraging, and normalising these self-regulatory skills:

*“I would come to my breath*,* and inhale deeply*,* and I’d say I invite you all to inhale deeply with me*,* inhale*,* exhale*,* and we do it three times.” (G9)*.

Affect regulation was particularly important in groups with high levels of trauma, in which dissociation and distress often occurred. Teachers used an engaged posture (e.g. turned towards, open, and relaxed), affirming body language (e.g. nodding) and words, which communicated acknowledgment, normalising and acceptance of what was arising for their participants.

### Being embodied: the foundation of body awareness in teaching and practising mindfulness

Awareness of the body was a dynamic, momentary experience – the knowing of surface sensations, those deeper within, as well as posture and position in space (intero-, extero- and proprio-ception). The body became a foundation on which regulation could unfold in a palpable way, the teacher modelling this capacity in the initial stages of a process and implicitly and explicitly inviting participants to explore this for themselves. Respondents described how being aware of their own somatic experience (and returning to this when noticing distraction) made the invitation to participants to notice bodily sensations and feelings, more real and accessible, and as such, more authentic. They felt that this tended to offset the tendency to abstract or reify experiences rather than knowing them directly:

*“(A) strong core element that really made a difference in terms of sensitive adaptation*,* was my own embodiment*,* a permissioning*,* an allowing rather than a strict imposition of anything. So how you were with people who were entering and who didn’t want to enter or to enter with trepidation*,* your embodiment was absolutely central to that. A kind of embodiment of an acceptance of the way people were.” (G2)*.

The guiding towards and the support of participants’ inhabiting their somatic experience, was infused with the attitudinal qualities of mindfulness in a trauma-sensitive way. Graduates focussed especially on deep attentiveness to subtle signs of distress with patience and acceptance – including when being in the body was not possible. This quality of acceptance was normalised, implicitly inviting people to try again, and bypassed the risk of shaming and activating self-judgment. The language used was often simple (e.g. short sentences), which were quite specific regarding what was being asked. It was also noted that sometimes the use of the mother tongue language appeared to be more effective in supporting participants to attend to their landscape of bodily experience:

*“I know that it’s landing when that happens*,* like when that comes from them they will suddenly forget about English…they just go into Xhosa*,* that’s when I’m like okay*,* they are here*,* it’s like they are in touch with what’s happening like here in the body right now.” (G4)*.

The value of paying attention to body posture and position sense, and the attitudinal quality infused by this, was noted. This was perceived to be amplified when working with refugees, given the quality and nature of their traumatisation:

*“When you deal with people like refugees who have lost their seat*,* they have been pushed. Everything around that seat is actually very powerful. So this idea I’m seeing you in your seat*,* it’s important for you to try to see and sense yourself in your seat as the starting blocks. In this position of dignity*,* with your head held high*,* chest open.” (G1)*.

### Adaptations to form, practice, and process

Adaptations to structure, form, and length of (formal) practices were considered and planned for prior to teaching. This was complemented by the need to adapt in any given moment to what was emerging within a session. Generally adapted forms were shorter in duration (6–8 weeks), length of session (90–120 min) and formal practices (3–30 min). In the case of the latter, participants were often offered a range of time to practice, and encouraged to explore and if possible, increase the time, if that felt possible as the programme progressed. The attitudes of mindfulness were prioritised within this context to ensure the integrity of the practice was retained.

A common theme was the value and importance of using techniques and tools that supported nervous system regulation. Breathing techniques, for example, were observed to be especially valuable to cope with high levels of dissociative tendencies and physiological hyperarousal, and to support grounding, alongside and integrated with brief mindfulness practices.

While in certain contexts the course was taught wholly in a local African language, in many others, teachers made use of a hybrid language field, in which words, phrases and some interactions were conducted in first languages if that was possible (i.e. the teacher was conversant in that language), alongside English. This appeared to enhance engagement with, understanding of and the capacity to embody mindfulness. By contrast, there were contexts in which the teacher used English exclusively, despite that being a second language for participants, where the focus was primarily on the attitudinal qualities being conveyed through non-verbal means, such as ritual and tone of voice. Many teachers found that storytelling, which is a very accepted part of African culture, along with metaphors and images, was an effective way to communicate core themes:

*“Some spoke very quickly in Afrikaans so that I didn’t always pick up every word. And I found it more expedient to thank them without a reflection but not to first ask for a translation… unless it was something I thought that I needed to get the actual clarity on. So I didn’t always reflect specifically*,* because I hadn’t actually heard what is going on. But I read his body language and I could feel where he was going and after my comments I would always invite them to respond as well: Have I said like you felt it?” (G10)*.

Teachers learnt that even with well thought-through structural adaptations, they often needed to let go what they had planned and simply flow with what was emerging in the group. This required being participant-centred, trusting, sensing and responding to what was arising in any given moment:

*“We said bring it into your walking (to and from school) as you walk those long distances. Feeling into the soles of your feet*,* feeling how it feels when you lift your leg and put it down. And when you arrive home*,* as you sit*,* do a little mini body scan*,* and see how it feels to finally sit on a chair.” (G9)*.

## Discussion

### Summary of key findings

**Fig. 1:**
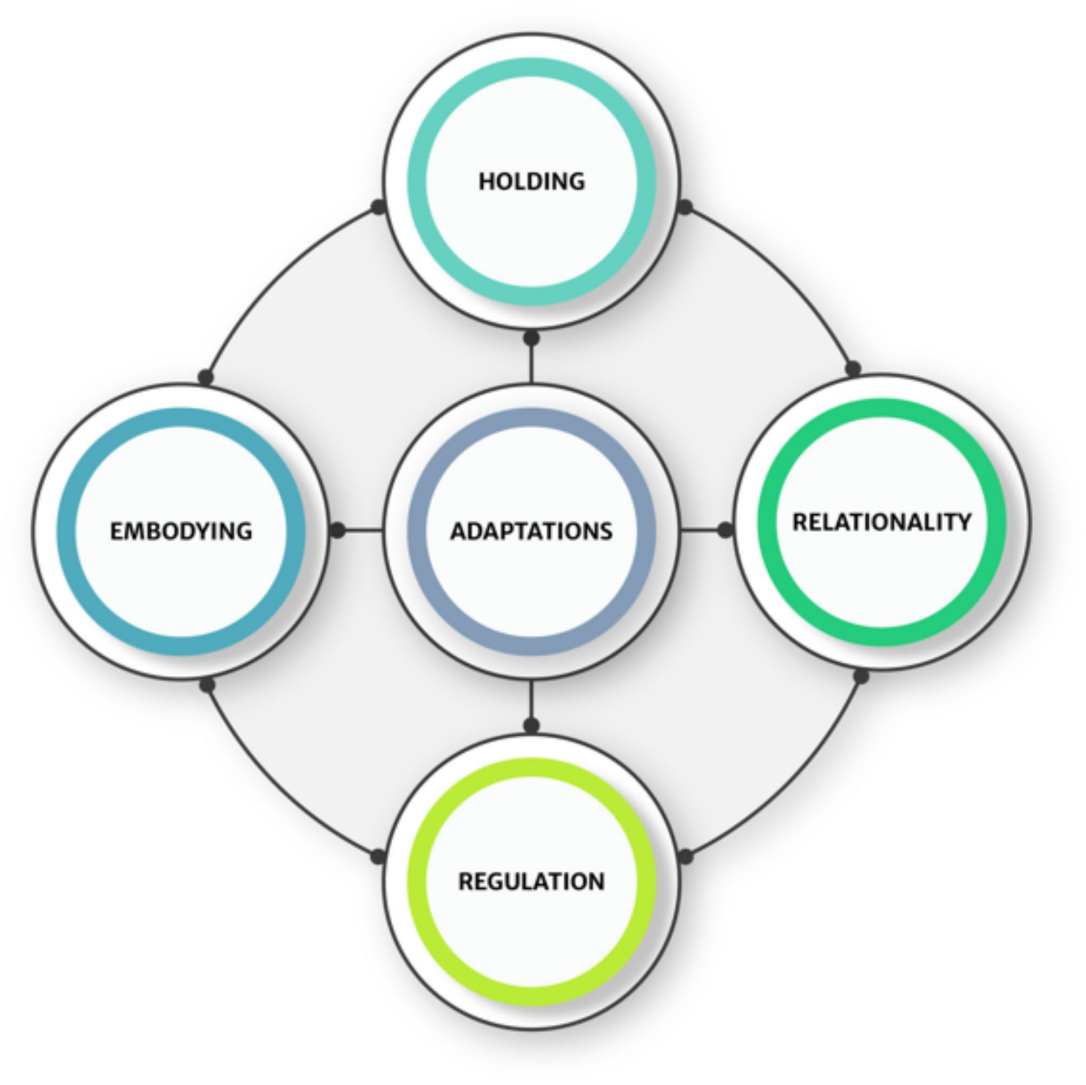
Context sensitive mindfulness: pillars of pedagogy

The key findings are summarised in Fig. 1. The model is a distillation of the core themes into five pillars, which reflect a dynamic and mutually reinforcing nest of interrelationships. Each pillar is either based on, informed by or infused with mindfulness, understood as an underlying way of being. Four of the pedagogic pillars for teaching mindfulness in the South African context were fundamentally inter-related: holding, relationality, regulation and embodying. The creation of a safe holding environment was foundational and incremental, based on the intentional cultivation of trusting and consistent relatedness. Within this framework, regulation could be intentionally introduced, both through trauma-sensitive mindfulness practices, and other practices that regulate the nervous system. These are not traditionally incorporated into MBIs. The capacity for teachers to engage with the group and to guide them in practice needed to arise from an embodied place to be most effectively internalised by participants. The felt sense of embodiment, that characterised a MBI teacher, was especially important in this context, as it generated immediacy and authenticity, and enhanced trust and relationality. Within this holding space, context-sensitive adaptations emerged in any given moment, over and above the prepared modifications, in a way that was sensitive and responsive to both the immediacy of experience and the implicit impact of context on that experience.

The initial observation, amongst those who taught adapted MBIs in communities, beset by psychological, social and economic stress, was that mindfulness is a potentially valuable practice, and can support people to manage their circumstances (internal and relational) more skillfully and kindly. It revealed at the same time that there is tremendous complexity in bringing mindfulness into many communities across the country. In particular, contextual issues related to language differences, access to basic resources, educational levels and unemployment, needed to be carefully considered before teaching began, as well as during and post the MBI. The personal and interpersonal manifestation of many of the dynamics was in the form of trauma. Thus, trauma sensitivity was an essential element to be considered before, during and after sharing mindfulness in communities and required a high level of sensitive atunement to be able to adapt to this in any given moment while teaching. This provided the context in which these pillars are located.

### Discussion of key findings

The MBI teacher assessment criteria (TAC) offers a framework for the training of teachers [41]. It provides theory and practical application for trainers, supervisors and learners in the developmental arc of becoming and maturing as a teacher of contemporary mindfulness [41–43]. The model describes six domains:

#### Domain 1

Coverage, pacing and organisation of session curriculum.

#### Domain 2

Relational skills.

#### Domain 3

Embodiment of mindfulness.

#### Domain 4

Guiding mindfulness practices.

#### Domain 5

Conveying course themes through interactive inquiry and didactic teaching.

#### Domain 6

Holding the group learning environment.

The pillars in Fig. 1 map generally onto these domains, but with two significant differences. Firstly, regulation is an additional pillar which was particularly important in the environment described in this paper, although may also be considered useful in other contexts. Secondly, context-sensitive adaptation to structure, form and content of mindfulness practices and approaches was a central feature and necessary requirement in such a trauma-dense environment.

All the MBI: TAC domains need to be intentionally trauma-informed as they shape an evolving local training curriculum. The one pillar not described in the MBI: TAC is the role of regulation. Training should foreground theoretical understanding of the role of mutual-regulation in the development of the capacity for self-regulation. This mirrors healthy early attachment dynamics, rather than trauma based attachment dysregulation [44, 45] and generates the basis for security priming [46]. This also potentially includes practices which are not classical mindfulness, such as breathing techniques, which have been shown to influence the regulation of the nervous system [47].

There is a growing body of research from within the mindfulness field, which recognises the significance of trauma, and the role that mindfulness-based approaches and trauma-sensitive mindfulness can play in the amelioration of trauma symptoms [48–51]. These findings, drawn from those teaching mindfulness in trauma-dense local communities, revealed foundational elements which need to be integrated and implemented at all levels of mindfulness-based pedagogy in South Africa.

The findings here reveal and explore traumatic experiences that clearly had an impact on individual and social functioning, although the long-term effects on resilience and functionality cannot be determined. Most of the findings are from people living at the lower end of the socio-economic continuum and, therefore, are from a context characterized by impoverishment and limited resources, both material and psychological, and which affects a majority of people across the country, mostly along racial lines [52].

In South Africa, the reality for many communities is continuous traumatic stress (CTS) [53] in contradistinction to the ‘post’ in posttraumatic stress disorder (PTSD). This more accurately describes the reality of psychological and social dynamics associated with living in an environment of ongoing threat, the “circumstances of which are inescapable” [54]. Research suggests that CTS may evoke a distinct response pattern that is different from PTSD. For example, hypervigilance is commonly experienced, but is functional in contexts of ongoing violence and threat [53]. It is self-evident, therefore, that the training of mindfulness-based teachers in the local context should always be trauma-informed. This may affect the duration of practices, use of language, appreciation of trauma dynamics and the risk of re-traumatisation [39, 50, 54].

One potential way to begin to mitigate risk, based on the current findings, is to create a milieu that is more likely to serve the cultivation of embodied presence “exposing individuals to stimuli designed to activate a sense of love, comfort, and safety… creating a sense of security similar to that induced by the presence of supportive others” [55]. This will naturally involve regulating the nervous system, and the soothing system and parasympathetic nervous system in particular. The former is considered a “primary-process core affect (which) arises from ancient subcortical processes” [56, 57]. The latter involves upregulation of the myelinated ventral fibres of the vagus nerve (the most phylogenetically recent), and is associated with relational processes which settle, engage and connect the individual in a prosocial field of relationality. This is self-soothing and calming, and orientates the physiology to a state of regulation and restoration [58]. The up-regulation of these neurophysiological systems is central to a safe holding environment and is conducive to the generation of a felt-sense of compassion, including towards oneself, as this can only emerge from and within an experience of safety [59].

The literature in South Africa suggests that traumatic stress is both an individual and broader social issue. Kanthamoney and Eagle suggest that mindfulness interventions in this and related global contexts must be “flexible in implementation and appropriate for contexts of single, multiple and continuous traumatization” [54]. They further argue that “community responsive intervention(s) in relation to both single event and ongoing trauma exposure… [should] reclaim some of the egalitarian and compassion enhancing aspects of mindfulness as a way of being and set of related practices” [54]. The kind of pedagogy that informs such interventions must take this imperative into account, informing both the content and qualitative dimensions of the curriculum. These qualities that lie at the heart of mindfulness emerged as one of the strongest themes in this cohort of graduates. In particular kindness and compassion characterised the way in which these teachers considered, held and responded to their participants. They embodied warmth and relationality, patience and care, providing a supportive environment for their participants to learn mindfulness. Taken together, an emerging context-congruent curriculum would be based on the five described pillars, and infused with compassion. This will allow for flexibility in form and structure, as well as sensitive, skilful, in-the-moment adaptability, when sharing mindfulness practices and mindfulness-informed interventions in diverse communities in this region.

### Limitations and future directions

The data analysis reached thematic saturation among interviewees who implemented mindfulness in impoverished settings, highlighting recurring themes. The first author’s (SW) close proximity to the course material and participants offered both advantages and limitations. Familiarity with the topic allowed for richer data collection and more substantive interpretation. The first author had developed relationships with the participants, based in mindfulness and compassion, and characterised by open honest communication. This also mitigated the potential for social desirability bias. The analysis was also conducted under the supervision of the second author (RM) who was not immersed in the training course and implementation of MBIs.

Qualitative studies on mindfulness have been criticized for their reliance on first-person data to assess efficacy and mindfulness practices [60]. However, given the novel nature of the experiences in this study, a qualitative approach was deemed appropriate. Future studies could validate these findings through second-person research that triangulates participant experiences and through third-person data using cross-sectional surveys with a representative sample of teachers.

The innovative work of graduates who introduced mindfulness practices in challenging environments is foundational to the evolving pedagogy of mindfulness-based approaches in LMICs like South Africa. Effective adaptations of the training curriculum require consideration of delivery systems that best serve local communities and empower those working within these environments [54]. Although repurposing mindfulness-based interventions to benefit underserved communities may be both feasible and desirable, it is essential to acknowledge the challenges inherent in delivering community-oriented mindfulness-based approaches with limited resources. The development of such training should be grounded in a pedagogical framework that integrates trauma-informed and trauma-sensitive approaches across all curriculum aspects, including theory, relationships, and practice.

One criticism in this field is the limited societal reach of mindfulness-based approaches, which are predominantly accessible to middle-class individuals, thereby excluding much of the South African population [61]. The number of people affected by stress and trauma far exceeds the availability of trained professional service providers, particularly in communities with the greatest needs [62]. Consequently, training innovation must extend beyond professionals to include non-professionals already working in these communities to enhance reach, scalability, relatability, and affinity.

Arising out of these imperatives, a proposed emergent training curriculum will feature two tiers instead of the current single-tier model. The second tier will continue in its existing form, while the new first tier will focus on sharing trauma-sensitive mindfulness and compassion-based practices in a broader range of contexts. This will involve shorter, adaptable practices with a strong emphasis on trauma sensitivity and relational support. Collaborating with non-profit organizations that offer mental health programmes and employ many non-professional care workers with an established presence in communities will support scalability and reach. These findings are now informing revisions to the current training in South Africa, aiming to equip graduates with the skills needed to bring these transformative practices to a broader range of South and Southern Africans in a way that enhances access and contextual relevance.

## Data Availability

The data that support the findings of this study are available from the corresponding author, SW, upon reasonable request.

## Declarations

### Use of artificial intelligence

No AI tools were used in the writing of this manuscript.
